# Collection of Data on Persons Living With Dementia Who Go Missing: First Responder Perspectives

**DOI:** 10.1093/geroni/igab046.2426

**Published:** 2021-12-17

**Authors:** Noelannah Neubauer, Serrina Philip, Samantha Dawn Marshall, Christine Daum, Hector Perez, Antonio Miguel-Cruz, Lili Liu

**Affiliations:** 1 University of Waterloo, Waterloo, Ontario, Canada; 2 University of Waterloo, University of Waterloo, Ontario, Canada; 3 University of Waterloo, Edmonton, Alberta, Canada; 4 University of Alberta, Edmonton, Alberta, Canada

## Abstract

While it is commonly cited that 60% of persons living with dementia (PLWD) wander, it is unclear whether this number reflects global contexts. Population aging has created a pressing need for the development of programs to mitigate the risks of PLWD from getting lost and going missing. Such programs would require a national strategy for the collection and integration of data on missing incidents involving this population. This study is a first step to inform such a strategy. The purposes were to: 1) identify approaches to data collection on missing persons incidents involving PLWD among Canadian police and search and rescue (SAR) organizations; 2) describe the foreseeable challenges associated with developing a national data collection strategy. We used generic qualitative description to generate data with fifteen key informants. Virtual semi-structured interviews were completed and transcribed verbatim. Content analysis and trustworthiness strategies guided analysis and rigor. Our findings indicate that police and SAR organizations collect a multitude of data pertaining to missing incidents involving PLWD. However, there is a lack of standardization in data collection, entry and analysis. Privacy legislation, limited resources, and incompatible data management systems pose challenges to data sharing and interoperability. Underreporting of missing incidents to police results in an underestimation of missing incidents. An intersectoral, uniform approach to data collection would enable the storage, analysis and comparison of national data. Accurate data on critical wandering can inform prevention, search strategies, resource allocation and effectiveness of programs.

